# Tutors´ and Students’ Agreement on Social and Cognitive Congruence in a Sonography Peer-assisted-learning Scenario

**DOI:** 10.1007/s40670-023-01814-y

**Published:** 2023-06-09

**Authors:** Ivo Rollmann, Jan Lauter, Charlotte Kuner, Anne Herrmann-Werner, Till J. Bugaj, Hans-Christoph Friederich, Christoph Nikendei

**Affiliations:** 1 Department for General Internal Medicine and Psychosomatics, University Hospital Heidelberg, Thibautstraße 4, 69115, Heidelberg, Germany; 2 Clinical Ultrasound Course at Medical Faculty, University of Heidelberg, Heidelberg, Germany; 3 Medical Department VI/Psychosomatic Medicine and Psychotherapy, University Hospital Tübingen, Tübingen, Germany; 4 Medical Faculty Tübingen, Tübingen Institute for Medical Education, Tübingen, Germany

**Keywords:** Peer-assisted learning, Clinical skills, Social and cognitive congruence, Sonography, Medical education

## Abstract

**Purpose:**

Peer-assisted learning has become an integral part within medical education and has been proven to be effective in teaching medical skills. Cognitive and social congruence are important factors that explain the effectiveness of peer-assisted learning. However, although theory suggests this, there is no study to date that demonstrates that students and tutors agree upon the level of cognitive and social congruence. Thus, we compared tutors’ and students’ perception of cognitive and social congruence and their agreement on the causes of congruence.

**Methods:**

36 students and 9 tutors from 9 courses were asked to answer questionnaires for their perception of cognitive and social congruence in a peer-assisted learning sonography scenario.

**Results:**

Students and tutors experienced cognitive congruence (t = 0.8277, df = 8, p = .4318, 95% CI = [-0.232; 0.491]) and social congruence (t = 0.962, df = 8, p = .364, 95% CI = [-0.145; 0.354]) similarly. In contrast, students and tutors disagreed on causes of cognitive congruence (agreement = 53.90%) and social congruence (agreement = 58.49%). Tutors rated their empathy and interest toward students as the main cause. Students rated the helpfulness, effectiveness, and approachableness of the tutor as the main cause.

**Conclusions:**

Our study filled the gap in previous research on cognitive and social congruence. Consistent with theoretical considerations, it was shown that students and tutors do indeed experience cognitive and social congruence similarly. Nevertheless, differences also emerged that may carry more or less weight depending on the research question. Future studies should therefore carefully examine whether the assessment of cognitive and social congruence of students and tutors is necessary.

## Introduction

Peer-assisted learning has become an integral part of most modern medical curricula [1] and is characterized by medical students teaching their peers. Hereby, either medical students of the same year (same year peer teaching) or medical students in higher years (cross-year peer teaching) teach other medical students as tutors [1]. Peer-assisted learning is most commonly used to teach students basic clinical skills, such as diagnostics, pediatrics, surgery, and internal medicine [2]. Especially in emergency medicine and intensive care units, peer-assisted learning is appreciated, as many students can participate in simulation-based training simultaneously. A popular example is cardiopulmonary resuscitation [3]. Another preferred practice of peer-assisted learning is the teaching of communications skill and interview techniques [4]. Some niche applications exist too, such as reducing the anxiety or stress of medical students before exams [5], helping international students to better integrate socially [6], and improving the learning experience during medical clerkships on wards [7].

Acceptance of peer-assisted learning is generally high and students sometimes prefer their peers over faculty staff as teachers [1, 8]. Peer-assisted learning courses are proven to increase the students’ self-confidence in performing basic clinical skills, such as blood withdrawal, peripheral venous catheter insertion, and diagnostic interviews [7, 9]. Regarding objective performance measures, students taught by their peers significantly improve their competencies in clinical skills, such as cardiac ultrasound [10], laparoscopy [11], and resuscitation [3]. Further examination also reveals differences between peer-assisted learning and faculty staff teaching: While two systematic reviews [12, 13] found no differences between these two forms of teaching for most clinical skills, other studies showed that faculty staff teaching is superior when it comes to more complex skills, such as emergency echocardiography [14] and spinal manipulation skills [15].

One possible explanation for the effectiveness of peer-assisted learning lies in a high cognitive and social congruence between students and tutor’s, which fosters the learning experience [16]. Cognitive congruence is described as the similar knowledge base of students and tutors. Social congruence is defined as students and tutors sharing similar social roles [16]. Cognitive congruence can be seen, for example, in the fact that students and tutors use the same language and prefer informal communication. Social congruence, in turn, can be recognised by the fact that, on the one hand, the students experience the tutor as supportive, and, on the other hand, the tutor allows plenty of time for the students' questions. Especially the tutors’ cognitive congruence seems to be one reason for the effectiveness of peer-assisted learning [17]: As they can relate to various task-based problems they themselves have already mastered, tutors are particularly empathic towards their fellow students. Finally, in a scoping review, Loda et al. [18] confirmed that cognitive and social congruence ‘represent relevant key factors in the peer-assisted learning context.’

Thus, the review of the literature illustrates that cognitive and social congruence are interpersonal factors that can compensate for the difference in knowledge, allowing tutors to teach as effectively as faculty staff to a large extent. Despite this, no study to date has operationalised cognitive and social congruence as an interpersonal factor. All studies cited thus far assessed cognitive and social congruence by surveying students only and therefore treated it as an intrapersonal factor. We know of only one study that assessed cognitive and social congruence by surveying tutors [19]. However, students were not interviewed in this study. In other words, although previous research formulates cognitive and social congruence as interpersonal factors, these are operationalised as intrapersonal factors. Therefore, we want to address this gap. Our aim is to measure the agreement between the cognitive and social congruence recorded by students and tutors. While the theory and conceptualisation of cognitive and social congruence as an interpersonal construct suggests that there should be a high agreement between students and tutors, there is in fact no evidence of this in previous research. In addition, the literature also suggests that students and tutors agree on the causes of cognitive and social congruence. Therefore, it is assumed that there is a high agreement between students and tutors in regard to cognitive and social congruence, as well as on the causes.

## Methods

### The Clinical Ultrasound Course at Medical Faculty, University of Heidelberg (CLUE)

The Clinical ultrasound course CLUE is a student initiated, organized, and executed facultative offered to medical students during the internal medicine module of the clinical part (3rd to 5th year) of the Heidelberger Curriculum Medicinale “HeiCuMed” [20]. The CLUE courses are an additional and voluntary offer made by the university and are held once a semester. CLUE consists of three modules. The first two modules focus on the basics of abdominal sonography, as well as the technical basics of duplex sonography. Each module includes two two-hour slots. Groups consist of four students and one peer tutor. The tutors and the students remain in the same constellation throughout the three modules.

### Medical Education in Germany

In Germany, medical training takes place in two sections. The first section is called the preclinical section and lasts 2 years. Students then move to the clinical section until the end of their training. Within the preclinical section, students learn all the theoretical medical basics. In the clinical section, medical students start with internships on different wards to practice medically relevant skills. CLUE courses therefore take place at the start of the clinical section, in order to help students learn sonography skills.

### Study Design and Participants

This study was designed as an exploratory cohort study. We surveyed all students who decided to participate in a CLUE course in October 2020. Participation in the study was voluntary. Tutors were employed to teach one medical skill course consisting of four students. In total, we surveyed 9 CLUE courses, resulting in a sample of 9 tutors and 36 students. All respondents took part in the study.

### Tutor Training and Education

The courses are based on peer-assisted learning and taught by medical students that are between their 3rd and 5th year. Tutor training consists of four peer-coaching weekends, where new tutors are trained in technical skills, didactics, and theoretical knowledge by senior tutors. To ensure a sufficient training of new tutors, their first pre-clinical sonography course is held in so-called “tandems”. First, a new pre-clinical tutor observes a senior tutor teaching a class and then proceeds to teach a class himself, whilst the senior tutor watches and assists. After a successful first pre-clinical course, tutors can join the CLUE team. During another peer-coaching weekend, the new CLUE tutors are taught the necessary practical skills, theoretical background, and clinically relevant pitfalls for the respective modules. All senior CLUE tutors must attend one peer-coaching weekend per year in order to ensure quality and consistency of the courses.

### Sample Size

We aimed at measuring the students’ and tutors’ agreement on cognitive and social congruence. Being the first study to measure such an agreement, we had to estimate its size. Theory and previous studies implied that medical students and tutors were highly cognitive and socially congruent [18]. This led us to believe that there would be a high agreement between medical students and tutors. Our study design paired four medical students and one tutor into one group, thus having five observations for one group. We calculated the need for at least three groups in order to find a high agreement (r = 0.8) against the null hypothesis of no agreement using the approximation of Walter et al. [21]. We were able to increase the sample to nine groups, which allowed us to accurately identify even medium interrater agreements.

### Ethics

This study received approval from the Ethics Committee of Medical Faculty Heidelberg (No. S-765/2020) in November 2020. The participation was on voluntary base and all medical students and tutors provided their written informed consent.

### Assessment and Measurements

Medical students and tutors were both asked to answer one composition of questionnaires. This composition consisted of questionnaires about sociodemographic data, cognitive and social congruence and several psychometric variables, and was answered after the medical skills course.

#### Sociodemographic Information

We asked medical students and tutors, for their age, gender, their year of study, and possible work experience they had before studying medicine. Tutors were further asked how long they have been tutors, which tutor trainings they have attended, and which medical skills courses they have taught.

#### Cognitive and Social Congruence

We captured medical students’ and tutors’ experienced cognitive and social congruence using the *social and cognitive congruence questionnaire* [22]. This questionnaire measures cognitive and social congruence on an observable behavioural level by asking about the tutors’ behaviour. The items range on a scale from 0 = ‘I strongly disagree’ to 4 = ‘I strongly agree’. To generate a version for tutors the word ‘tutor’ was changed to ‘I’ for each item. For Item 21 (‘It calms me, that the tutor successfully passed the course.’) no adaption could be created. Cognitive congruence is measured with 7 items and resulted in a Cronbach’s Alpha α = 0.78 for our sample. Social congruence is measured with 14 items for the tutors and 15 items for the students and had a α = 0.91 in our sample.

#### Psychometric Variables

We asked both medical students and tutors to answer three questionnaires for psychometric variables in order to assess personality and attachment styles as possible confounding variables. First the *BFI-10,* a short version of the Big Five Inventory that assesses the five personality traits within one minute [23], was used. Each personality trait is measured with two items on a 5-point Likert scale from 1 = ‘strongly disagree’ to 5 = ‘strongly agree’. Cronbach’s alpha for extraversion, conscientiousness, agreeableness, openness and neuroticism were α = 0.86, 0.34, 0.31, 0.24 and 0.50 respectively. Second, the *OPD-SF KV,* a screening instrument to assess a person’s structural integration of personality, was used. Such aspects included are the ability to perceive oneself, the ability to get into contact with another person, and the inner representations of relationships [24]. All three of those personality dysfunctions are measured with four items on a 5-point Likert scale from 0 = ‘does not apply at all’ to 4 = ‘is completely true’. We used the total score for our analysis, which achieved a α = 0.77 in our sample. Third, we used the *RQ2* developed by Bartholomew and Horowitz [25] to assess four prototypic attachment styles: Secure, preoccupied, dismissing, and fearful attachment. Each of these attachment styles is measured with one Item on a 7-point Likert scale from 1 = ‘Not applicable at all’ to 7 = ‘Very strongly applicable’.

### Statistical Analysis

We conducted all calculations in the R environment [Version 4.0.5; 26].

#### Confounding Variables

We ran bivariate correlations to assess if cognitive and social congruence were confounded with our psychometric variables.

#### Comparison of Students’ and Tutors’ Perception

To test for a difference in the students’ and tutors’ perception of cognitive and social congruence, we first calculated the average mean rating for all students in a course and the mean rating of the tutor. Then a t-test for paired samples was conducted to test if the differences of the ratings significantly differ from 0. A significant result represents a significant difference between the perceived congruence of students and tutors. This analysis was conducted separately for both cognitive and social congruence.

#### Agreement on Causes of Congruence

Each item of the *cognitive and social congruence questionnaire* [22] refers to one aspect of the tutors’ behavior that is responsible for social or cognitive congruence. In other words, if a student or tutor believes that one aspect of the tutors’ behavior was the cause for the cognitive and social congruence he will rate the corresponding item high. We used those ratings to calculate the students’ and tutors’ agreement on causes of cognitive and social congruence. Afterwards, we averaged the agreements within one medical skills course and then across all medical skills courses as proposed by Döring and Bortz [27]. We repeated this procedure for both cognitive and social congruence. Based on general consensus, we treated an average agreement across all medical skills courses of over 0.8 as a strong agreement that supported our hypothesis [28].

As implied by previous studies, we expected medical students and tutors to consistently rate cognitive and socially congruent high [18]. Such skewed distributions bear the risk of the kappa paradox, wherein raters mostly use the same category, yet still reach low interrater reliability and agreement [29]. To counter the kappa paradox, we estimated the agreement using polychoric correlation, percentage of agreement, and percentage of agreement, allowing for a difference of one point. Polychoric correlation is based on the idea that raters actually perceive a latent variable as continuous but are forced to rate it categorically. Polychoric correlation then tries to extrapolate the correlation between the latent continuous variables from the categorical ratings [30]. It can be shown that polychoric correlation correctly estimates the agreement, while other interrater coefficients over- or underestimate the agreement between raters [31].

## Results

### Confounding Variables

We found no significant correlations with possible confounding variables.

### Comparison of Students’ and Tutors’ Perception of Cognitive and Social Congruence

#### Cognitive Congruence

**Fig. 1:**
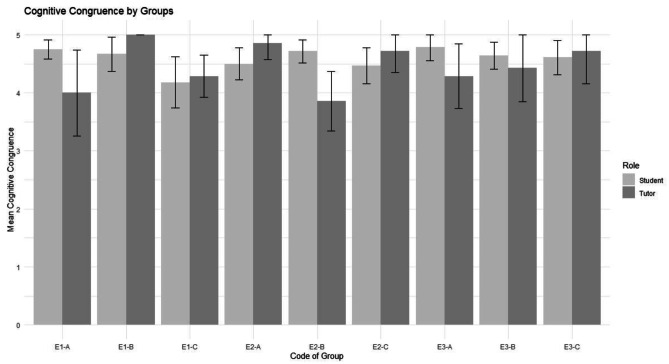
Cognitive Congruence of medical students and tutors per medical skills course. Error bars represent 95% confidence intervals

As seen in Fig. 1., medical students and tutors perceive high cognitive congruence. Most tutors rating of cognitive congruence equals the medical students’ ratings as seen by the 95% confidence intervals in Fig. 1. A paired sample t-test revealed no significant difference between the tutors’ and medicals students’ perception of cognitive congruence: t = 0.8277, df = 8, p = 0.4318, 95% CI = [-0.232; 0.491].

#### Social Congruence

**Fig. 2:**
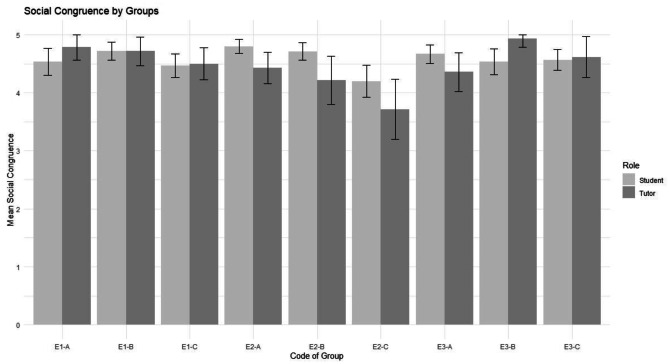
Social Congruence of medical students and tutors per medical skills course. Error bars represent 95% confidence intervals

Similar results could be seen with social congruence. As seen in Fig. 2, tutors and medical students perceive high social congruence. Most tutors’ rating of cognitive congruence equals the medical students’ ratings as seen by the 95% confidence intervals in Fig. 2. A paired sample t-test revealed no significant difference between medical students’ and tutors’ perception of social congruence: t = 0.962, df = 8, p = 0.364, 95% CI = [-0.145; 0.354].

### Students’ and Tutors’ Agreement on Causes of Congruence

#### Cognitive Congruence

There was a low polychoric correlation between medical students’ and tutors’ ratings, averaging at r_pol_ = 0.242, as seen in Table 1. Furthermore, the percentage of agreement was at an average of 53.90%. Percentage of agreement with a tolerance of 1-Point difference averages at 88.10%. In other words, in 34.20% of all items the ratings of students and tutors differed by one point.

**Table 1:** Interrater reliability of cognitive congruence

Code of Group	Polychoric Correlation	Agreement in Percent	Agreement in Percent (1 Point Tolerance)
E1-A	0.520	28.57	85.71
E1-B	NA	81.55	89.29
E1-C	0.334	42.86	85.71
E2-A	0.364	71.43	85.71
E2-B	0.421	53.57	89.29
E2-C	-0.279	53.57	89.29
E3-A	-0.110	39.29	85.71
E3-B	0.343	42.86	92.86
E3-C	0.341	71.43	89.29
Average	0.242	53.90	88.10

Annotation: In group E1-B, several students only rated 5, which disallowed calculating a polychoric correlation

#### Social Congruence

Similar results could be seen for social congruence. As seen in Table 2., average polychoric correlation was low at r_pol_ = 0.457, with a percentage of agreement at 58.49%. Allowing for a tolerance of 1-point difference raised percentage of agreement to 92.45%. It other words in 33.96% of all items the ratings of students and tutors differed by one point.

**Table 2:** Interrater reliability of social congruence

Code of Group	Polychoric Correlation	Agreement in Percent	Agreement in Percent (1 Point Tolerance)
E1-A	0.270	64.29	94.64
E1-B	0.722	73.21	96.43
E1-C	0.800	55.36	94.64
E2-A	0.774	55.36	98.21
E2-B	0.486	66.07	92.86
E2-C	0.591	21.43	71.43
E3-A	0.338	51.79	92.86
E3-B	-0.679	69.64	92.86
E3-C	0.813	69.23	98.08
Average	0.457	58.49	92.45

### Descriptive Analysis

As can be seen in Table 3, although students and tutors perceive social and cognitive congruence the same on average, they differ in their ratings on specific items. Purely descriptively, we observed that tutors place a primary emphasis in their ratings on the interest and empathy one shows students. On the other hand, students placed more value on the tutor's ability to help, effectiveness, and approachability.

**Table 3:** Social and Cognitive Congruence questionnaire for Students and Tutors

Items	Students		Tutors	
	Mean	SD	Mean	SD
Same knowledge base *	4.08	0.84	3.89	1.05
Similar language *	4.67	0.68	4.33	0.71
**Preferring informal contact** *	4.14	1.10	4.44	0.88
Student wasn’t afraid to tell tutor if they didn’t understand anything *	4.78	0.72	4.44	0.53
Being interested in students’ needs and problems **	4.75	0.55	4.56	0.73
**Helping students** **	4.89	0.40	4.78	0.44
Tutor was able to explain students the topics based **	4.86	0.49	4.67	0.50
Taking time for questions **	4.83	0.56	4.56	0.73
**Supportiveness of student tutor** **	4.83	0.56	4.78	0.44
Showing empathy **	4.83	0.45	4.89	0.33
Being interested in student as learner **	4.72	0.61	4.78	0.44
Effectiveness of the student tutor **	4.42	0.77	4.00	0.71
Being open and approachable **	4.89	0.40	4.78	0.44
**Helpful and constructive feedback** **	4.42	0.77	3.75	0.89
Seeing tutor as role model **	3.89	0.85	3.67	0.87
Stress-free and relaxing learning atmosphere *	4.83	0.45	4.78	0.44
Being interested in students **	3.89	1.01	4.33	0.50
Easy and informal communication *	4.81	0.47	4.67	0.50
Trustful learning base **	4.69	0.62	4.56	0.53
Creating open and non-judgmental learning environment *	4.83	0.45	4.67	0.50
Successfully passed the course **	3.86	1.29	**/**	**/**
Effectiveness of tutorial **	4.86	0.42	4.44	0.73

Annotation: * = Item of cognitive congruence, ** = Item of social congruence. Successfully passed the course could not be recoded to a student version

## Discussion

Although in previous research cognitive and social congruence have always been conceptualized as interpersonal factors, in the implementation of the studies, they have been operationalized as if they were an intrapersonal factor. While theory suggested that students and tutors agree on cognitive and social congruence, there was no evidence yet that this is actually the case. To fill this gap, we were the first study to measure student-tutor agreement on cognitive and social congruence in a sonography course. At the item level, student and tutor responses differed by more than 1 point on a 5-point Likert scale in only 10% of cases. Furthermore, the descriptive evaluation of the items showed that students and tutors set slightly different weightings in their ratings. Tutors placed more emphasis on the interest and empathy one shows to students in their responses. In contrast, students placed more emphasis on the tutor's helpfulness, their effectiveness as a teacher, and how easy it was to ask them questions. In other words, tutors rated emotional and motivational factors as the main cause of cognitive and social congruence, while students rated behavioural aspects as the main cause.

There are several possible explanations for our results: The study was conducted within a peer-assisted sonography learning scenario, which pairs one tutor with four medical students. While sufficient for our research purpose, this is a rather small group, allowing much interaction between students and tutors. Consequently, it is possible that the perceived high social and cognitive congruence is caused by the highly interactive scenario. Second, nearly all students and tutors highly agreed with the items of the questionnaire. This led to a highly skewed distribution. Thus, tutors may indeed have been cognitive and socially congruent, which the ratings confirm. Yet, from a perspective of measurement theory, such high ratings might be the consequences of a ceiling effect [27]. A ceiling effect would imply that students and tutors should have had prior training in using the *social and cognitive congruence questionnaire* [22]. Another implication of a ceiling effect would concern the questionnaire itself: The items ask whether one agrees that the tutor acted congruently. In other words, the questionnaire differentiates between congruent and non-congruent behaviour, but allows no further differentiation. This would explain the high ratings and equal distribution of agreement and high agreement. Döring and Bortz [27] suggest the use of anchor examples or changing the rating scale to counter such ceiling effects. Self-assessment and external assessment may be another explanation for the disagreement of students and tutors. The items can logically be categorized into questions asking about the tutors’ emotional states, the students’ perceptions of the tutor, and about the learning environment. Thus, the tutor can directly perceive their own emotional states, but has to guess the students’ perception, and vice versa. The difference in ratings may represent the self-assessment and external assessment, as opposed to a disagreement on the causes of congruence.

### Limitations and Future Directions

Our results may be specific to our sample, may represent ceiling effects or may represent a systematic difference in assessment of students and tutors. In addition, our sample, although sufficiently sized for our hypothesis [21], consisted of only 9 tutors and 36 students, which might have influenced our results. Accordingly, future studies should test our results. One possible future direction would be a replication of our results with different samples and within different peer assisted learning scenarios. Another possible direction would be evaluating the psychometric properties of the *social and cognitive congruence questionnaire* [22]. Other questions raised by our study concern the learning scenario and the role of social and cognitive congruence: As seen in literature, peer-assisted learning is effective in teaching medical skills to small and big groups of students and social congruence is thought to be a key factor in explaining its effectiveness [3, 4, 12, 16, 18]. As we discussed, one possible reason for the high perceived social and cognitive congruence may be our small group size. As a result of this, it could be questioned if peer-assisted learning may be less effective with bigger groups, as it may lead to lower social and cognitive congruence. More broadly, it remains unclear whether social and cognitive congruence is a necessary requirement for effective peer-assisted learning to occur or a supporting condition that leads to more effective peer-assisted learning. With regard to these considerations, future studies should systematically alter the group size or instruct the tutors to control the perceived social and cognitive congruence and subsequently evaluate the effectiveness of the peer-assisted learning scenarios.

## Conclusions

Although cognitive and social congruence have been conceptualized as interpersonal factors in previous research, they have only been operationalized as intrapersonal factors. Previous research assessed cognitive and social congruence by surveying only students or only tutors. Although theory suggests that students’ and tutors’ assessments of cognitive and social congruence should be similar, there has been no evidence of this. Our study filled this gap by being the first to measure student-tutor agreement on cognitive and social congruence within a sonography peer-assisted learning scenario. The results coincided with the theory. Students and tutors agreed on cognitive and social congruence. However, students attributed cognitive and social congruence to different causes than tutors. Tutors rated emotional aspects to be the main cause of congruence, while student rated the tutors’ help, effectiveness, and approachableness to be the main causes of congruence. Future studies can therefore assume that students and tutors mostly agree on their social and cognitive congruence. For more specific questions, however, it is worthwhile to capture both perspectives in order to work out the precise differences.

## Practice Points

- This is the first study that directly compares students’ and tutors’ social congruence.
- Students and tutors seem to perceive the same social and cognitive congruence between them.
- Students and tutors disagree on the causes of congruence.
- Tutors rate their interest in and empathy for the students as the main cause of congruence.
- Students rate the helpfulness and approachableness of the tutor as the main cause of congruence.

## Data Availability

All data may be requested by contacting the first author.
